# Application value of FRAX in high-altitude areas in China: a systematic review and meta-analysis

**DOI:** 10.3389/fendo.2026.1656377

**Published:** 2026-02-13

**Authors:** Yong Guo, Ying Zhang, Ting-Ting Jiao

**Affiliations:** 1 Department of Hospital, Daxing Teaching Hospital, Capital Medical University, Beijing, China; 2 Department of Oncology, Daxing Teaching Hospital, Capital Medical University, Beijing, China; 3 Department of Traditional Chinese Medicine, Daxing Teaching Hospital, Capital Medical University, Beijing, China

**Keywords:** fracture risk, FRAX, high altitude, meta-analysis, osteoporosis

## Abstract

**Background:**

High-altitude environments in China are associated with altered bone metabolism and an increased risk of osteoporotic fractures. Given the limited availability of dual-energy X-ray absorptiometry (DXA) in these regions, the FRAX tool provides a practical alternative for fracture risk assessment. However, the screening performance and diagnostic performance of FRAX in plateau populations remain unclear. This study systematically evaluated the diagnostic accuracy of FRAX—probability of major osteoporotic fracture (PMOF), probability of hip fracture (PHF), and FRAX high-risk thresholds—in high-altitude areas in China.

**Methods:**

Six English and Chinese databases were searched from inception to December 31, 2025. Studies reporting true-positive, false-positive, false-negative, and true-negative values for FRAX in populations residing above 1,500 m were included. Pooled sensitivity, specificity, likelihood ratios, diagnostic odds ratio (DOR), and area under the summary receiver operating characteristic (SROC) curve (AUC) were calculated using random-effects models. Heterogeneity was explored through meta-regression and subgroup analyses based on region, bone mineral density (BMD) inclusion, reference standard, gender, and study design. Sensitivity analyses excluding studies with high or unclear Quality Assessment of Diagnostic Accuracy Studies 2 (QUADAS-2) risk were performed.

**Results:**

Eleven studies were included. For PMOF, the pooled sensitivity and specificity were 0.70 and 0.82, respectively (AUC = 0.82), while PHF yielded pooled estimates of 0.68 and 0.84, respectively (AUC = 0.82). Both analyses showed substantial heterogeneity (*I*^2^ > 90%). Subgroup analyses indicated higher specificity in Qinghai–Tibet Plateau populations and mixed-gender cohorts. Meta-regression identified region, BMD inclusion, reference standard, and gender as contributors to heterogeneity. Sensitivity analyses excluding lower-quality studies produced similar estimates. Only three studies evaluated the FRAX high-risk threshold, and the extremely wide confidence intervals—particularly for specificity (95% CI: 0.32–0.98)—indicated marked imprecision.

**Conclusions:**

FRAX may provide preliminary and exploratory information for fracture risk screening in high-altitude settings; however, the evidence remains limited and imprecise, precluding firm conclusions regarding its clinical usefulness.

**Systematic review registration:**

https://inplasy.com/wp-content/uploads/2025/01/INPLASY-Protocol-7296.pdf, identifier INPLASY202510040.

## 1 Background

Osteoporosis (OP) is a systemic metabolic disease characterized by decreased bone mass and bone structure destruction, leading to reduced bone strength and increased risk of osteoporotic fractures (OFs) (1). Due to its insidious onset and high mortality and disability rates (2), how to better predict fracture risk and provide timely intervention has always been a focus of clinical research. The Centre for Metabolic Bone Diseases at Sheffield University launched the FRAX tool in 2008, which helps predict the 10-year risk of hip or other major site osteoporotic fractures (3). It is simple to use and has good generalizability. It has been validated in untreated patients aged 40 to 90 years from four races in Europe and the Americas (4). The osteoporosis diagnosis and treatment guidelines issued in China recommend FRAX for individuals aged 40 and above, using the American threshold criteria: a high risk of osteoporotic fracture is indicated when the major osteoporotic fracture (MOF) ≥20% or hip fracture (HF) ≥3% (5). However, due to differences in geography and race, the application of FRAX in different regions and populations in China requires further investigation.

The high-altitude areas in China (such as the Yunnan–Guizhou Plateau, Qinghai Plateau, Inner Mongolia Plateau, and Loess Plateau) are characterized by low oxygen, low pressure, and cold climate (6), making osteoporosis more likely to occur (7, 8). The review on the relationship between high-altitude environment and osteoporosis states that the high-altitude environment increases the incidence of osteoporosis (9). A Chinese multi-ethnic cohort study involving 99,556 research subjects showed that exposure to high altitudes may reduce adult bone density, thereby increasing the risk of osteoporosis (10). A retrospective study using China Health and Retirement Longitudinal Study (CHARLS) data involving 3,192 research subjects showed that exposure to high-altitude areas increases the risk of the most severe complications of osteoporosis, namely, hip fractures (11). Low pressure and hypoxia are regarded as the main causative factors affecting bone metabolism at high altitudes (12, 13). As the primary physiological stressors in the high-altitude environment, both of them can lead to abnormal bone remodeling (14). Hypoxia can affect the natural metabolism of bone tissue, disrupt the balance between bone resorption and bone formation (15), cause excessive proliferation of osteoclasts, damage the osteogenic potential of mesenchymal stem cells (16), and inhibit the differentiation of osteoblasts (17), thereby accelerating bone density loss, and may also induce oxidative stress damage, affecting bone metabolism (18). Therefore, conducting research on osteoporosis in high-altitude areas is of great significance.

Given the limited medical resources and low prevalence of bone density testing in high-altitude areas, the device-independent FRAX tool can compensate for resource shortages and significantly enhance the efficiency of early screening for osteoporotic fractures. The high-risk threshold of FRAX was originally designed for treatment decisions. In recent years, FRAX has been applied in clinical diagnostic analysis and has demonstrated excellent predictive performance. However, its predictive efficacy may be affected by altitude-related differences in bone metabolism. At present, there is no meta-analysis study on the application of FRAX in high-altitude regions.

This study aims to verify the screening performance of the FRAX tool in China’s high-altitude areas and provide evidence for optimizing local fracture risk assessment strategies.

## 2 Materials and methods

We followed the Preferred Reporting Items for Systematic Reviews and Meta-Analyses (PRISMA) guidelines in this systematic review. The review protocol has been registered with the International Platform of Registered Systematic Review and Meta-analysis Protocols (INPLASY), number INPLASY202510040 (https://inplasy.com/wp-content/uploads/2025/01/INPLASY-Protocol-7296.pdf).

### 2.1 Literature search

A comprehensive literature search was conducted across six electronic databases: PubMed, Embase, Web of Science, the Cochrane Library, China National Knowledge Infrastructure (CNKI), and Wanfang Data. The search spanned from the inception of each database to December 31, 2025, and was limited to studies published in English or Chinese. The search strategy was constructed using a combination of Medical Subject Headings (MeSH) and free-text terms. The core search terms included the following:

(“FRAX” OR “Fracture Risk Assessment Tool”) AND (“high altitude” OR “plateau” OR “elevation”) AND (“China” OR “Chinese”) AND (“osteoporosis” OR “bone density” OR “fracture”).

In PubMed, we applied a structured search strategy combining MeSH and free-text terms. The final search string was as follows:

(“FRAX”[Title/Abstract] OR “Fracture Risk Assessment Tool”[Title/Abstract])AND (“high altitude”[Title/Abstract] OR plateau[Title/Abstract] OR elevation[Title/Abstract])AND (China[Title/Abstract] OR Chinese[Title/Abstract])AND (osteoporosis[MeSH Terms] OR osteoporosis[Title/Abstract] OR “bone density”[Title/Abstract] OR fracture[Title/Abstract]).

Equivalent search terms were used for Chinese databases. In addition, the reference lists of all included articles and relevant reviews were manually screened to identify additional eligible studies. Gray literature, such as dissertations and conference proceedings, was also manually retrieved from CNKI and Wanfang. All identified records were imported into the Zotero (version 7.0.17; Corporation for Digital Scholarship, Vienna, VA, USA) software for duplicate removal.

### 2.2 Inclusion and exclusion criteria

Inclusion criteria: 1) The purpose of the study was to evaluate the diagnostic value of FRAX for severe osteoporosis or fracture. 2) There were clear diagnostic criteria for severe osteoporosis or osteoporotic fracture. The diagnostic criteria for osteoporosis (any one of the following three conditions must be met) were as follows: fracture of the hip or vertebra; dual-energy X-ray absorptiometry (DXA) measurement of T-value ≤ −2.5; and bone density measurement conforms to osteopenia (−2.5 < T-value < −1.0 for proximal humerus, pelvis, or distal forearm), where T-value ≤ −2.5 + fracture of fragility belongs to severe osteoporosis. 3) The study subjects were from high-altitude areas in China, defined as regions with an elevation above 1,500 m (19), including Qinghai, Tibet, Yunnan, Guangxi, Guizhou, Sichuan, Inner Mongolia, Shanxi, and Ningxia Hui Autonomous Region. 4) The true-positive (Tp) value, false-negative (Fn) value, false-positive (Fp) value, and true-negative (Tn) value of the 10-year probability of major osteoporotic fracture (PMOF) and the 10-year probability of hip fracture (PHF) can be directly (or indirectly) obtained.

Exclusion criteria: 1) Conference abstracts, case reports, preprints (not peer-reviewed), or studies that do not provide complete data were excluded; 2) studies with animals or cells as research subjects; 3) duplicate publications (identified by the same research team, sample, and results); and 4) systematic reviews or narrative reviews (non-original research).

### 2.3 Literature screening and data extraction

Two researchers in this study independently screened the literature, and inconsistencies were resolved by consulting a third-party expert. After reading the full text, relevant data were extracted, including the following: 1) first author and publication time; 2) region, ethnicity, gold standard, new standard, sample size, average age, and design type; and 3) outcome indicators: Tp, Fp, Tn, Fn, sensitivity, and specificity.

### 2.4 Quality evaluation

The quality evaluation was conducted using the diagnostic accuracy study quality evaluation method 2 [Quality Assessment of Diagnostic Accuracy Studies 2 (QUADAS-2)] list provided by the RevMan5.3 statistical software (20).

### 2.5 Statistical analysis

Statistical analyses were performed using Stata (version 15.0; StataCorp, College Station, TX, USA) and R (version 4.0.3; R Foundation for Statistical Computing, Vienna, Austria).

#### 2.5.1 Heterogeneity test

Using the Stata 15.0 software, the presence of threshold effects was judged by calculating Spearman’s correlation coefficient, and the heterogeneity caused by non-threshold effects was evaluated using the Q test and *I*^2^ test. If *p* > 0.1 and *I*^2^ < 50%, a fixed-effects model was used. If *I*^2^ ≥ 50% or *p* < 0.1, heterogeneity was considered to exist, and a random-effects model was adopted, and further meta-regression analysis was conducted to explore the sources of heterogeneity.

#### 2.5.2 Diagnostic efficacy evaluation indicators

The Stata 15.0 and RStudio 4.0.3 software were used to calculate the sensitivity, specificity, positive likelihood ratio (PLR), negative likelihood ratio (NLR), and diagnostic odds ratio (DOR); draw the summary receiver operating characteristic (SROC) curve; calculate the area under the curve (AUC); and draw the Fagan plot.

#### 2.5.3 Sensitivity analysis

Using the Stata 15.0 software, the stability of the results was tested by sequentially excluding literature. Based on the QUADAS-2 quality assessment, a sensitivity analysis was conducted using the “leave-one-out” method to verify the impact of low-quality studies on the pooled results. In addition to study-level covariates such as region (Qinghai–Tibet Plateau vs. others), reference standard type, study design, and gender distribution, we attempted to include clinically relevant continuous variables, such as mean age, body mass index (BMI), and bone turnover markers, which were pooled into the meta-regression analysis. However, due to inconsistent reporting and missing data across the included studies, these factors could not be incorporated in a quantitative fashion. This limitation may affect our ability to fully identify the sources of heterogeneity.

#### 2.5.4 Publication bias analysis

Using the Stata 15.0 software, Deeks’ funnel plot was drawn, with *p* < 0.05 indicating the presence of publication bias.

## 3 Results

### 3.1 Inclusion of literature results

**Figure 1:**
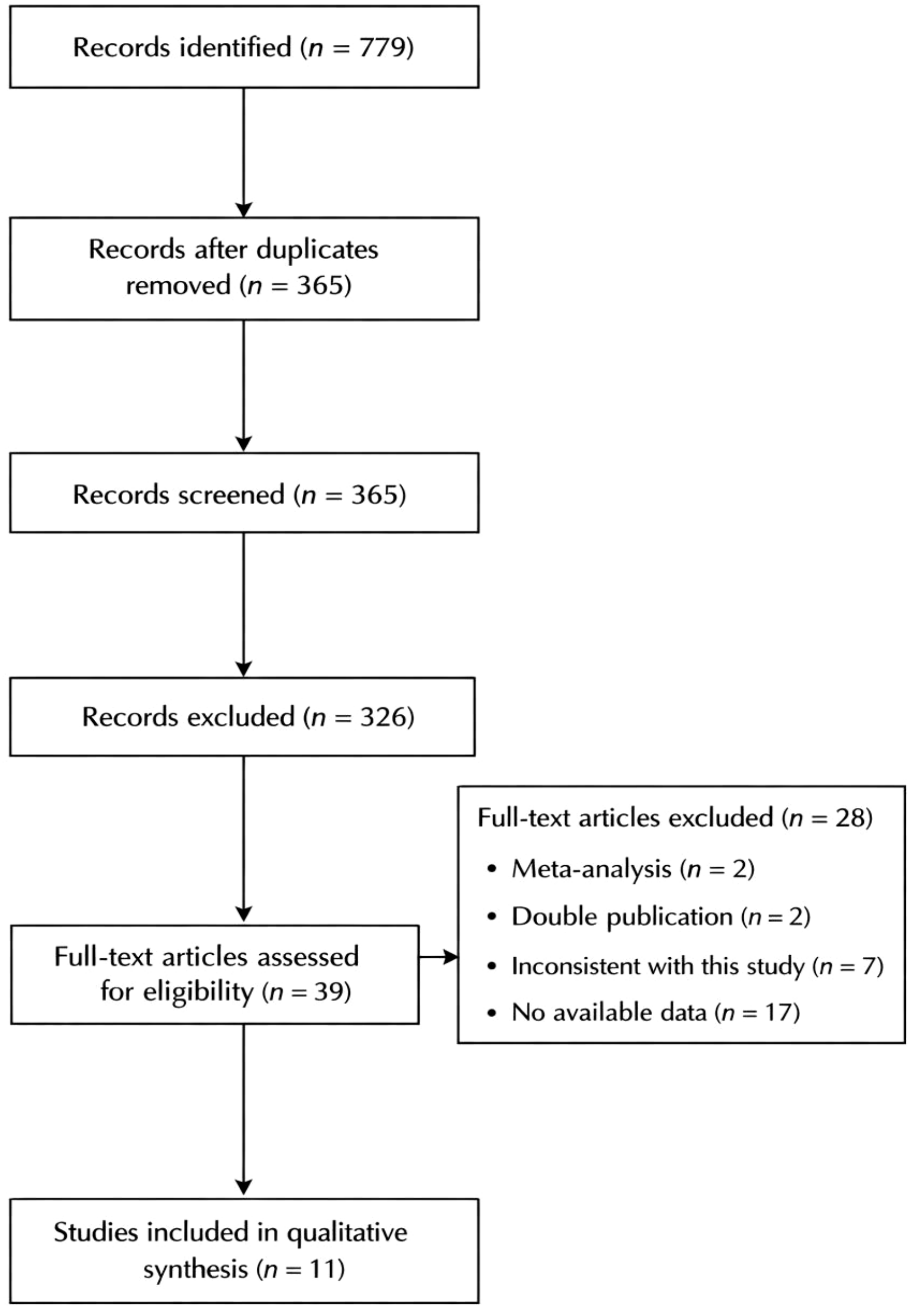
Literature search process and results. PubMed (n = 52), Cochrane Library (n = 7), Web of Science (n = 15), Embase (n = 20), China National Knowledge Infrastructure (CNKI) (n = 372), and Wanfang Data (n = 313).

A total of 779 related articles were initially searched, including 94 in English and 685 in Chinese. After step-by-step screening, 11 articles were finally included. The literature search process is shown in **Figure 1**.

### 3.2 Basic characteristics of included studies and QUADAS-2 quality evaluation results

**Figure 2:**
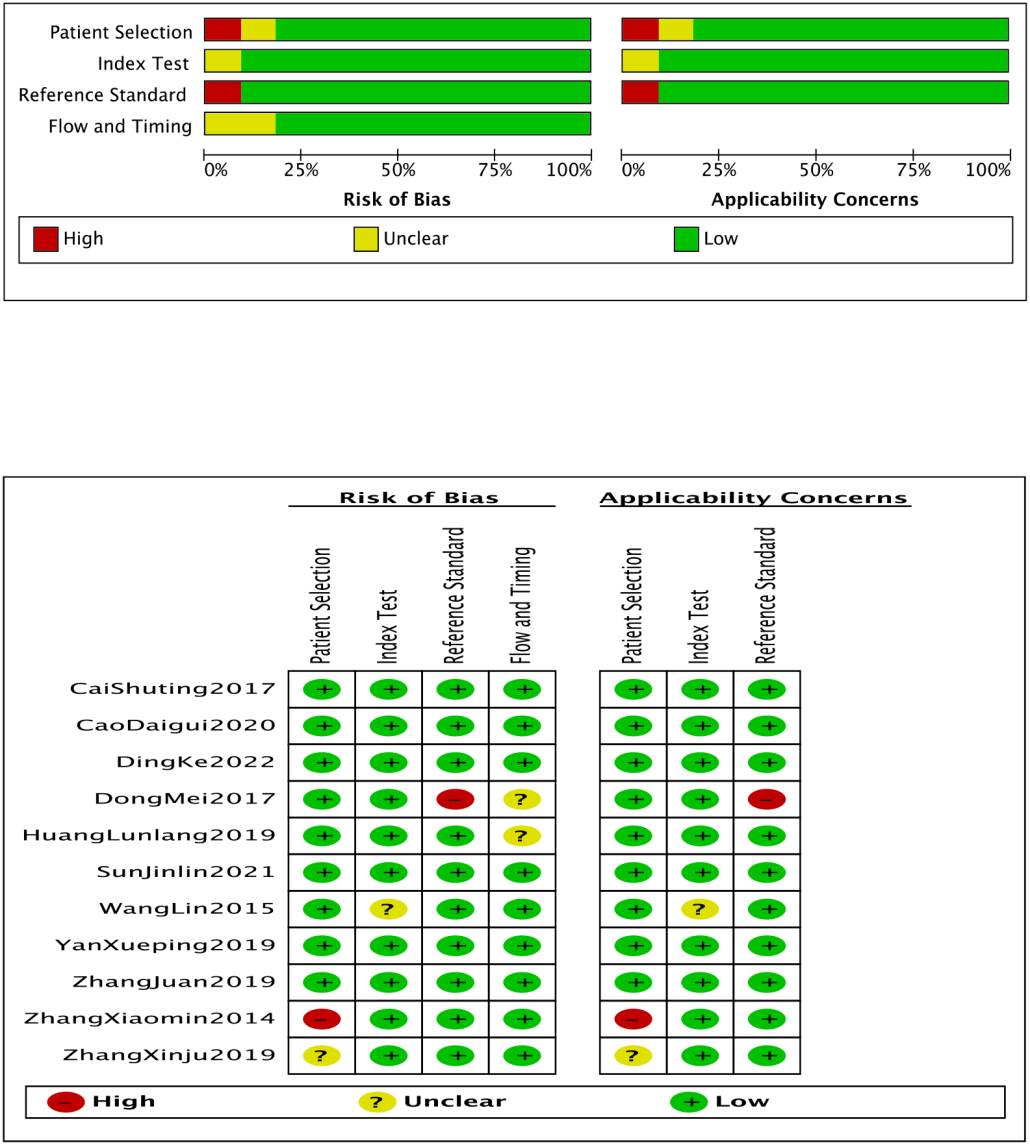
Quality evaluation results of literature.

The main high risks of the included studies were selection bias of the study subjects and bias of the gold standard. The basic characteristics of the included studies are shown in **Table 1**. The methodological quality of the 11 included studies was evaluated using the QUADAS-2 tool (**Figure 2**). Most studies were of acceptable quality, although some domains showed high or unclear risk.

**Table 1:** Basic characteristics of included literature.

Study	Region	Ethnicity	Gold standard	Sample size (n)	Age (years)	Gender	New standard	Study design	Tp	Fp	Fn	Tn	Se	Sp
Cai Shutong 2017 (21)	Guangxi	Zhuang	T < −1.0	166	≥50	Female	①	Cross-sectional	86	16	46	18	0.65	0.54
Zhang Juan 2019 (22)	Xinjiang	Han, Uyghur, Hui, Kazakh	Femoral neck bone density T-value ≤ −2.5	1,008	58.44 ± 9.86	All	⑤	Cross-sectional	59	23	24	902	0.71	0.98
Huang Lunlang 2019 (23)	Tibet	Han	Osteoporosis: (1) Presence of hip or vertebral fragility fracture; (2) DXA measurement of central bone density T-value ≤ −2.5; (3) −2.5 < T-value < −1.0 + proximal humerus, pelvis, or distal forearm fragility fracture, meeting any one of the above three	124	57. 67 ± 8. 86	All	②	Cross-sectional	3	0	21	100	0.13	1
							③		9	8	15	92	0.38	0.92
		Tibetan		128	55. 45 ± 10. 36	All	②		3	0	15	110	0.17	1
							③		10	12	8	98	0.56	0.89
Dong Mei 2017 (24)	Inner Mongolia	Han	T ≤ −2.5	316	61.1 ± 10.5	Female	②	Cross-sectional	16	129	5	166	0.767	0.563
							③		4	176	2	133	0.623	0.431
				63	62.7 ± 13.5	Male	②		2	34	2	26	0.562	0.431
							③		1	27	0	35	0.673	0.562
		Mongol		38	61.3 ± 11.9	Female	②		2	18	2	16	0.541	0.479
							③		3	19	1	14	0.673	0.431
				18	63.3 ± 13.6	Male	②		1	9	1	8	0.54	0.471
							③		2	8	1	6	0.601	0.423
Yan Xueping 2019 (25)	Sichuan	/	T ≤ −2.5	36	63.31 ± 10.14	Female	⑤	Cross-sectional	16	9	2	9	0.89	0.5
Cao Daigui 2020 (26)	Chongqing	/	Vertebra OF	287	>50	Female	②	Retrospective cohort	46	24	40	177	0.54	0.88
Zhang Xiaomin 2014 (27)	Shanxi	/	Hip OF	584	70 (54.3–83.0)	All	③	case control	237	41	55	251	0.81	0.86
							④	case control	134	15	158	277	0.46	0.95
Wang Lin 2015 (28)	Yunnan	/	OF	215	40–90	Female	⑤	Forward-looking cohort	11	68	5	134	0.69	0.66
Ding Ke 2022 (29)	Guangxi	Zhuang	OF	75	57.56 ± 11.12	All	②	Cross-sectional	12	17	2	44	0.86	0.72
							①	Cross-sectional	13	22	1	39	0.93	0.64
							④	Cross-sectional	2	9	2	62	0.5	0.87
							③	Cross-sectional	2	1	2	70	0.5	0.99
Zhang Xinju 2019 (30)	Xinjiang	/	OF	6000	71.28 ± 10.32	All	①	Retrospective cohort	2378	622	455	2545	0.84	0.8
Sun Jinlin 2021 (31)	Qinghai	Han, Hui, Tibetan	Femoral neck bone density T-value ≤ −2.5	324	/	All	①	Cross-sectional	26	28	0	270	1	0.91
							②	Cross-sectional	21	57	5	241	0.81	0.81
							③	Cross-sectional	26	9	0	289	1	0.97
							④	Cross-sectional	18	42	8	256	0.69	0.86

① PMOF with BMD (MOFT); ② PMOF without BMD; ③ PHF with BMD (HFT); ④ PHF without BMD; ⑤ FRAX high-risk: PHF ≥ 3% or PMOF ≥ 20%.

PMOF, probability of major osteoporotic fracture; BMD, bone mineral density; PHF, probability of hip fracture; Tp, true positive; Fp, false positive; Fn, false negative; Tn, true negative; Se, sensitivity; Sp, specificity; DXA, dual-energy X-ray absorptiometry; OF, osteoporotic fracture.

In the risk of bias domain, Zhang (27) was rated as high risk for patient selection, likely due to retrospective sampling. Dong et al. (24) showed a high risk in the reference standard and an unclear risk in flow and timing due to a lack of reporting on diagnostic blinding and consistency of patient follow-up. Wang (28) had unclear risk in the index test, suggesting insufficient detail on how FRAX was applied or interpreted. Zhang et al. (29) was rated unclear for patient selection. The remaining studies were rated as low risk across all domains.

In the screening performance concerns domain, Zhang (27) was judged to have high concern in patient selection, reflecting the limited representativeness of the population. Dong et al. (24) showed high concern in the reference standard and low concern in other domains. Zhang et al. (30) had unclear concerns in patient selection. Wang (28) had unclear concerns in the index test, suggesting possible inconsistencies in applying FRAX to the local population. All other studies were rated as low concern in all screening performance domains.

Overall, the included studies demonstrated a generally low risk of bias and good screening performance. However, findings from Dong et al. (24), Zhang (27), and Zhang et al. (30) should be interpreted with caution due to concerns in key methodological domains.

### 3.3 Meta-analysis results

#### 3.3.1 Application of PMOF in fracture risk assessment in plateau population

##### 3.3.1.1 Combined effect size

**Figure 3:**
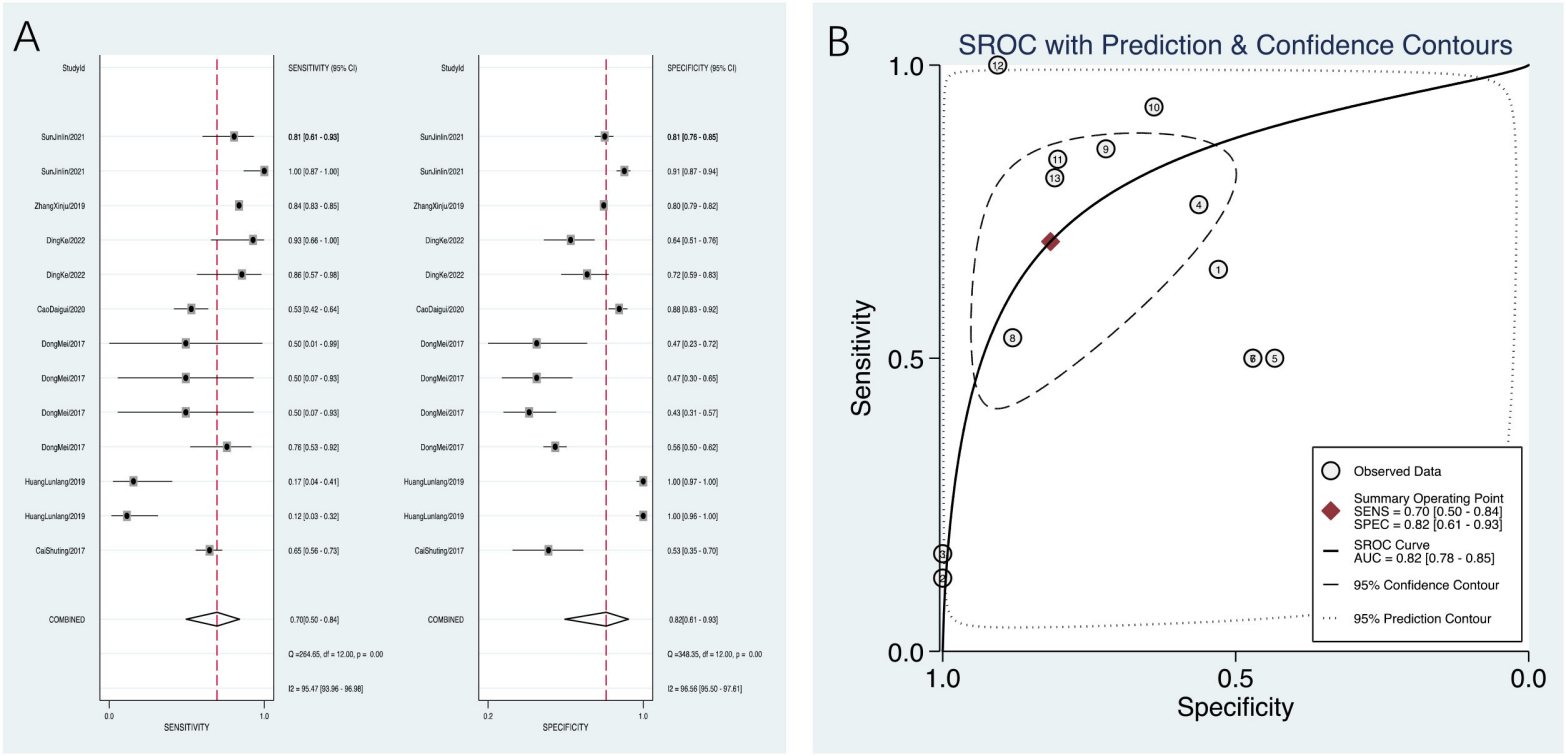
**(A)** PMOF forest plot. **(B)** PMOF SROC. PMOF, probability of major osteoporotic fracture; SROC, summary receiver operating characteristic.

A total of seven studies were included, with 13 subgroups. The results of Spearman’s correlation analysis showed that s = −0.09, *p* = 0.78, and there was no threshold effect, so the effect size could be combined for analysis. The heterogeneity analysis of sensitivity showed that *I^2^* = 95.47%, *p* = 0.00, and the heterogeneity analysis of specificity showed that *I^2^* = 96.56%, *p* = 0.00, both indicating significant heterogeneity, so a random-effects model was used. After combination, compared with the gold standard of severe osteoporosis or fracture, the sensitivity was 0.70 (95% CI: 0.50–0.84), the specificity was 0.82 (95% CI: 0.61–0.93), the PLR was 3.80 (95% CI: 1.80–8.00), the NLR was 0.37 (95% CI: 0.22–0.61), and the DOR was 10.00 (95% CI: 4.00–26.00) (**Figure 3A**).

##### 3.3.1.2 SROC curve drawing

The AUC value of PMOF predicting severe osteoporosis or fracture was 0.82 (0.78–0.85) (**Figure 3B**).

##### 3.3.1.3 Meta-regression

**Figure 4:**
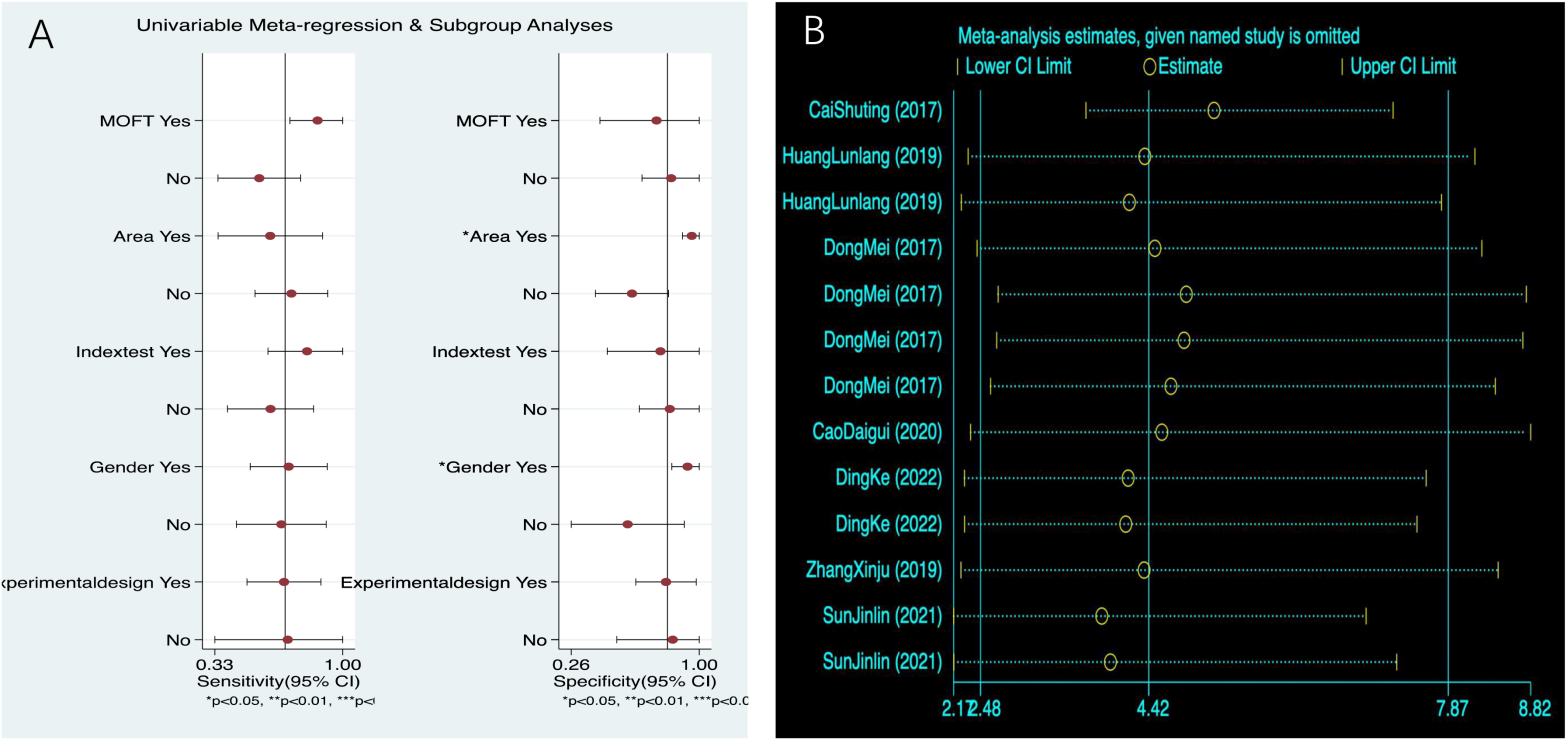
**(A)** PMOF meta-regression. **(B)** PMOF sensitivity analysis. PMOF, probability of major osteoporotic fracture.

According to the results of meta-regression, factors such as PMOF without BMD, Qinghai–Tibet Plateau area, not using fracture as the gold standard, not including all genders, and cross-sectional studies, the sensitivity of PMOF decreased; in PMOF with BMD, non-Qinghai–Tibet Plateau area, using fracture as the gold standard, not including all genders, and cross-sectional studies, the specificity decreased, all of which may be sources of heterogeneity (**Table 2**, **Figure 4A**). Overall, meta-regression indicated that BMD inclusion and fracture-based reference standards tended to increase sensitivity, whereas gender composition and regional differences contributed primarily to variability in specificity, consistent with the subgroup analysis findings.

**Table 2:** Meta-regression analysis of PMOF in fracture risk assessment in plateau population.

Parameter	Category	Number of studies	Sensitivity (95% CI)	P1	Specificity (95% CI)	P2
PMOF with BMD (MOFT)	Yes	4	0.87 (0.72–1.00)	0.12	0.75 (0.43–1.00)	0.71
	No	9	0.56 (0.35–0.78)	0	0.84 (0.67–1.00)	0
Qinghai–Tibetan plateau region (area)	Yes	5	0.62 (0.35–0.89)	0.42	0.96 (0.90–1.00)	0.01
	No	8	0.73 (0.54–0.92)	0	0.61 (0.40–0.82)	0
The gold standard is fracture (index test)	Yes	4	0.81 (0.61–1.00)	0.38	0.78 (0.47–1.00)	0.85
	No	9	0.62 (0.40–0.85)	0	0.83 (0.65–1.00)	0
Includes all genders (gender)	Yes	7	0.72 (0.52–0.92)	0.94	0.93 (0.84–1.00)	0.02
	No	6	0.68 (0.45–0.91)	0	0.59 (0.26–0.91)	0
Cohort study (experimental design)	Yes	2	0.71 (0.33–1.00)	0	0.85 (0.52–1.00)	0
	No	11	0.69 (0.50–0.89)	0.96	0.81 (0.63–0.98)	0.83

PMOF, probability of major osteoporotic fracture; BMD, bone mineral density.

##### 3.3.1.4 Subgroup analyses

###### 3.3.1.4.1 Subgroup analysis by region

**Figure 5:**
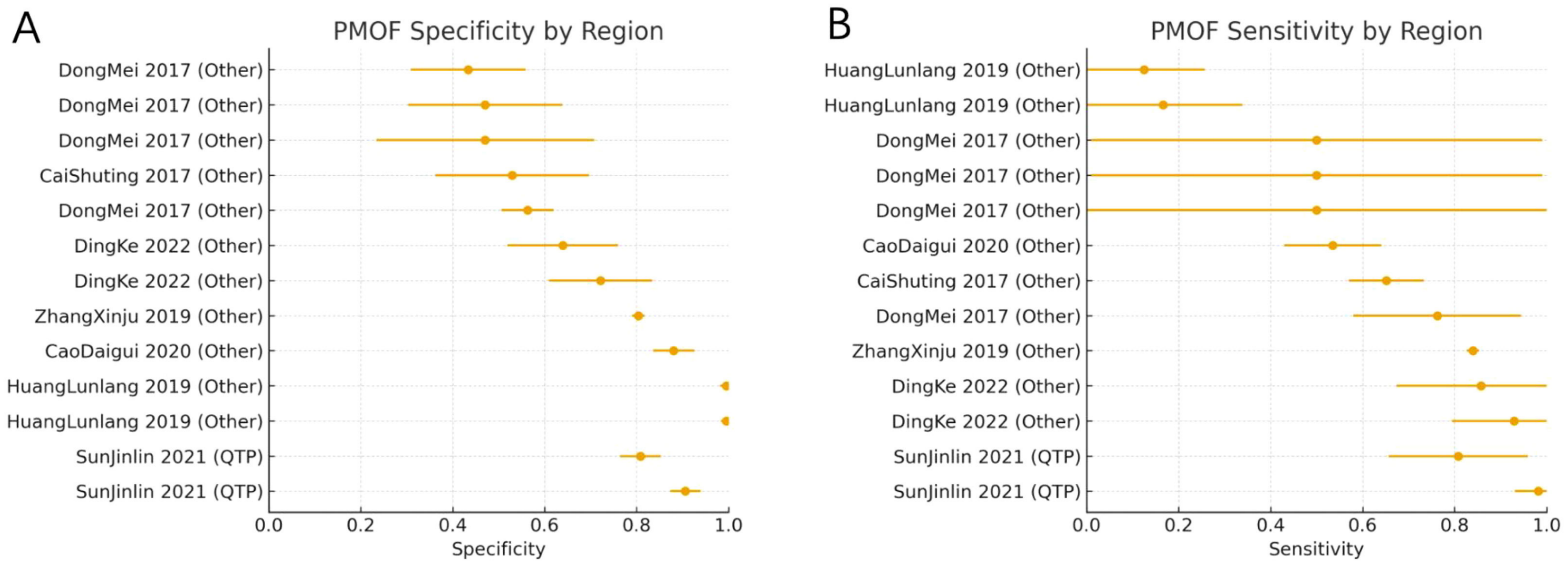
**(A)** PMOF specificity by region. **(B)** PMOF sensitivity by region. PMOF, probability of major osteoporotic fracture.

For PMOF, subgroup analyses by region showed that studies from the Qinghai–Tibet Plateau (QTP) had higher pooled sensitivity and specificity than those from other high-altitude areas. In non-QTP regions, the pooled sensitivity was 0.59 (95% CI: 0.43–0.76; *I*^2^ = 95.5%; k = 11), and the pooled specificity was 0.71 (95% CI: 0.62–0.79; *I*^2^ = 98.8%; k = 11). In contrast, studies conducted in the Qinghai–Tibet Plateau yielded a pooled sensitivity of 0.90 (95% CI: 0.72–1.00; *I*^2^ = 80.5%; k = 2) and a pooled specificity of 0.86 (95% CI: 0.76–0.95; *I*^2^ = 91.5%; k = 2). The forest plots of PMOF sensitivity and specificity stratified by region are shown in **Figure 5**.

###### 3.3.1.4.2 Subgroup analysis by gender

**Figure 6:**
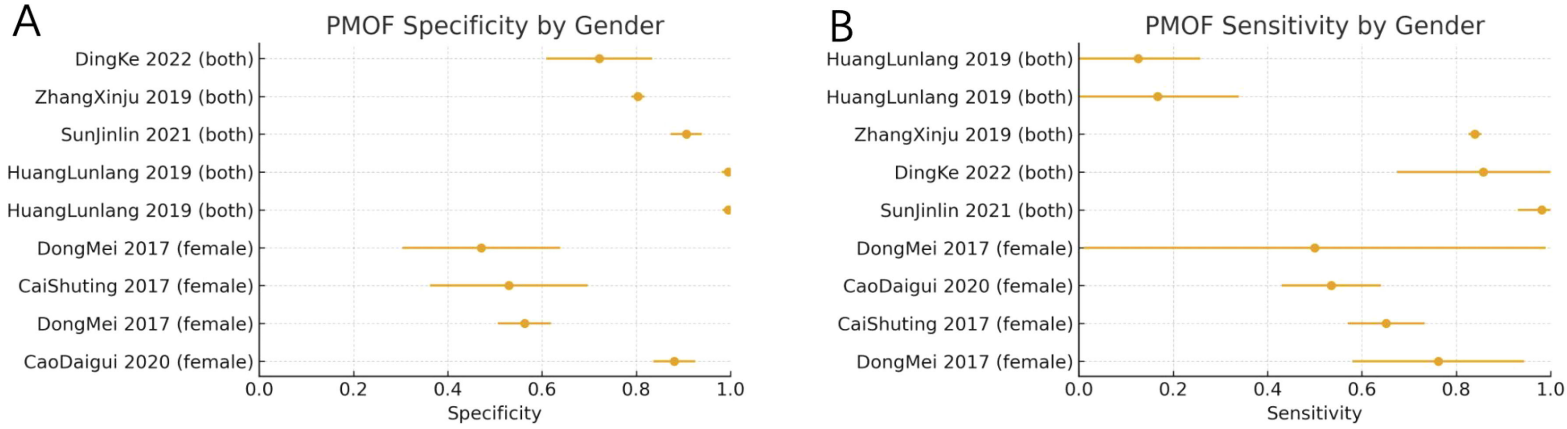
**(A)** PMOF specificity by gender. **(B)** PMOF sensitivity by gender. PMOF, probability of major osteoporotic fracture.

We further performed subgroup analyses for PMOF according to gender composition. In female-only cohorts, the pooled sensitivity was 0.63 (95% CI: 0.53–0.72; *I*^2^ = 46.7%; k = 4), and the pooled specificity was 0.62 (95% CI: 0.40–0.84; *I*^2^ = 96.7%; k = 4). In studies including both sexes, the pooled sensitivity was 0.61 (95% CI: 0.40–0.81; *I*^2^ = 97.8%; k = 5), and the pooled specificity was 0.89 (95% CI: 0.80–0.99; *I*^2^ = 99.2%; k = 5). Thus, including both men and women was associated with higher specificity but more variable sensitivity compared with female-only populations (**Figure 6**).

##### 3.3.1.5 Sensitivity analysis

Sensitivity analysis was conducted by sequentially excluding studies, and no study had a significant impact on heterogeneity, indicating stable results (**Figure 4B**). In addition, a sensitivity analysis excluding studies rated as high or unclear risk of bias in key QUADAS-2 domains (24, 27, 30) showed similar pooled estimates for both sensitivity and specificity, indicating that the overall results were not driven by lower-quality studies.

##### 3.3.1.6 Fagan plot

**Figure 7:**
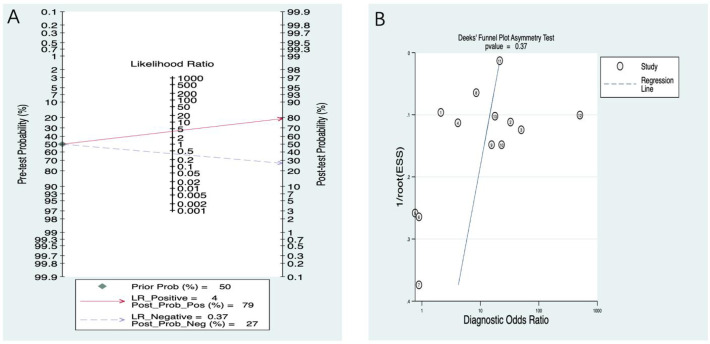
**(A)** PMOF Fagan. **(B)** PMOF publication bias. PMOF, probability of major osteoporotic fracture.

In the Fagan nomogram analysis (**Figure 7A**), the PLR was 4, and the NLR was 0.37. By connecting the pre-test probability with the values corresponding to the positive and negative likelihood ratios in the middle of the plot and intersecting with the post-test probability on the right, the post-test probability of positive and negative was obtained. Assuming that the initial correct identification probability of severe osteoporosis or fracture was 50%, the probability of a correct positive diagnosis through PMOF modeling prediction increased from 50% to 79%.

##### 3.3.1.7 Publication bias

The publication bias test result was *p* = 0.37 (95% CI: −15.21 to 2.17), indicating no publication bias (**Figure 7B**).

#### 3.3.2 Application of PHF in fracture risk assessment in plateau population

##### 3.3.2.1 Combined effect size

**Figure 8:**
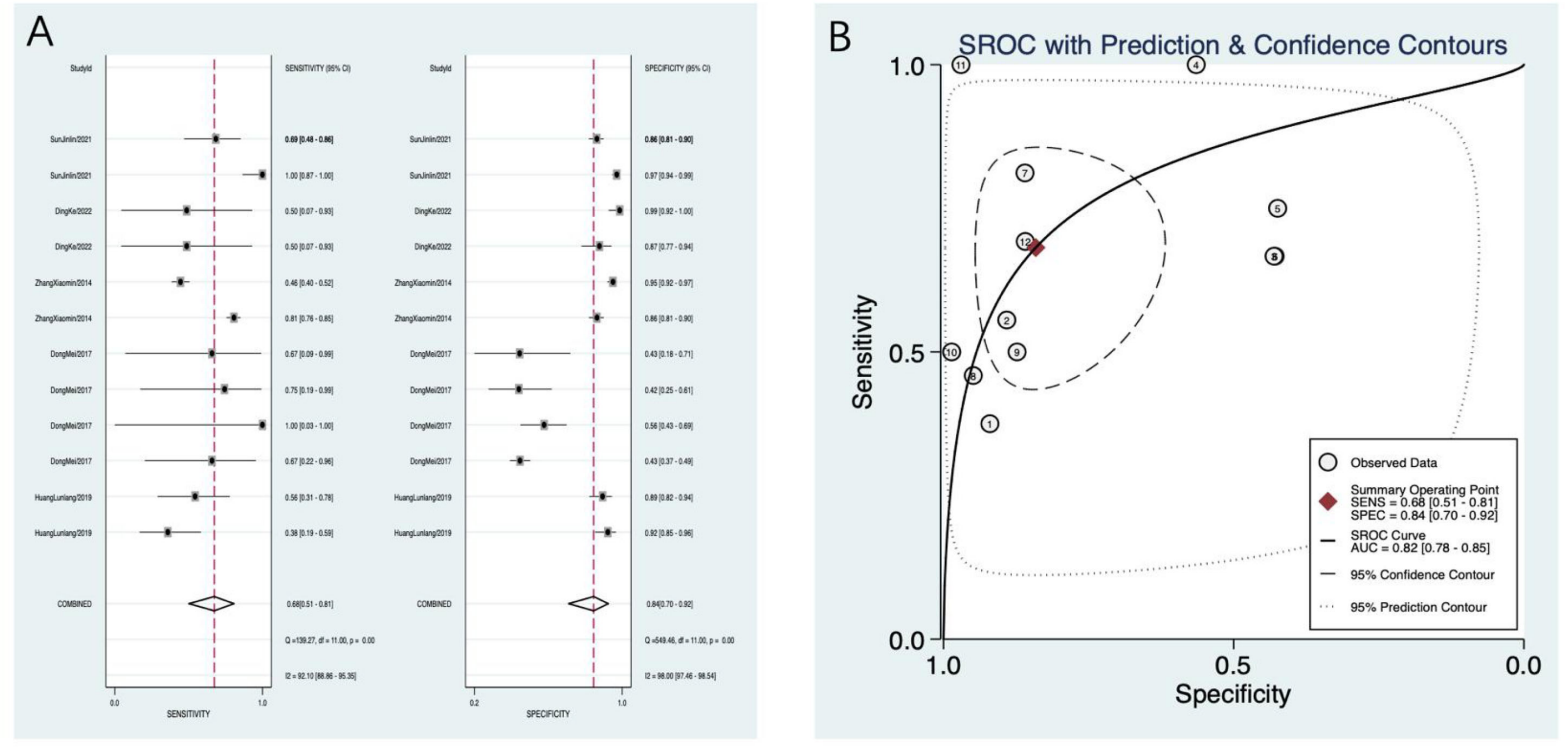
**(A)** PHF forest plot. **(B)** PHF SROC. PHF, probability of hip fracture; SROC, summary receiver operating characteristic.

Five studies were included, with 12 subgroups. The results of Spearman’s correlation analysis showed that s = −0.32, *p* = 0.31, and there was no threshold effect, so the effect size could be combined for analysis. The heterogeneity analysis of sensitivity showed that *I^2^* = 92.10%, *p* = 0.00, and the heterogeneity analysis of specificity showed that *I^2^* = 98.00%, *p* = 0.00, both indicating significant heterogeneity, so a random-effects model was used. After combination, compared with the gold standard of severe osteoporosis or fracture, the sensitivity of PHF was 0.68 (95% CI: 0.51–0.81), the specificity was 0.84 (95% CI: 0.70–0.92), the PLR was 4.30 (95% CI: 2.10–8.70), the NLR was 0.38 (95% CI: 0.23–0.62), and the DOR was 11.00 (95% CI: 4.00–33.00) (**Figure 8A**).

##### 3.3.2.2 SROC curve drawing

The AUC value of PHF predicting severe osteoporosis or fracture in plateau population was 0.82 (0.78–0.85) (**Figure 8B**).

##### 3.3.2.3 Meta-regression

**Figure 9:**
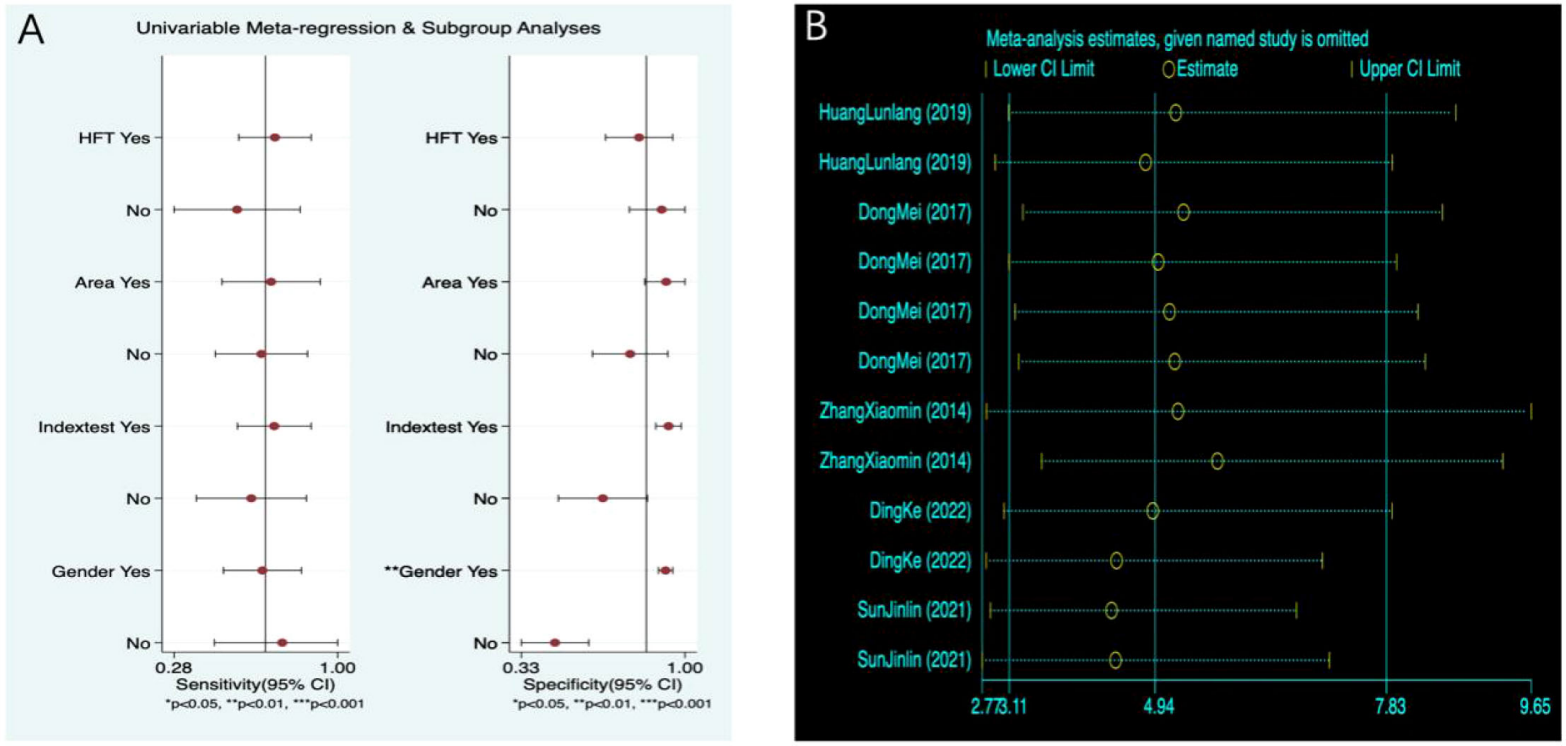
**(A)** PHF meta-regression. **(B)** PHF sensitivity analysis. PHF, probability of hip fracture.

According to the results of meta-regression, in PHF without BMD, non-Qinghai–Tibet Plateau area, not using fracture as the gold standard, and including all genders, the sensitivity of PHF decreased; in PHF with BMD, non-Qinghai–Tibet Plateau area, not using fracture as the gold standard, and not including all genders, the specificity decreased, all of which may be sources of heterogeneity (**Table 3**, **Figure 9A**). The direction of effects observed in the meta-regression was broadly consistent with the subgroup analyses, with region and reference standard contributing most strongly to specificity variation.

**Table 3:** Meta-regression analysis of PHF in fracture risk assessment in plateau population.

Parameter	Category	Number of studies	Sensitivity (95% CI)	P1	Specificity (95% CI)	P2
PHF with BMD (HFT)	Yes	9	0.72 (0.56–0.88)	0.52	0.81 (0.67–0.95)	0.31
	No	3	0.56 (0.28–0.83)	0	0.90 (0.77–1.00)	0
Qinghai–Tibetan plateau region (area)	Yes	4	0.71 (0.49–0.92)	0.93	0.92 (0.83–1.00)	0.37
	No	8	0.66 (0.46–0.87)	0	0.77 (0.62–0.93)	0
The gold standard is fracture (index test)	Yes	6	0.72 (0.56–0.88)	0.83	0.93 (0.88–0.98)	0.14
	No	6	0.62 (0.38–0.86)	0	0.66 (0.48–0.85)	0
Includes all genders (gender)	Yes	8	0.67 (0.50–0.84)	0.6	0.92 (0.89–0.95)	0
	No	4	0.76 (0.46–1.00)	0	0.47 (0.33–0.60)	0

PHF, probability of hip fracture; BMD, bone mineral density.

##### 3.3.2.4 Subgroup analysis by region

**Figure 10:**
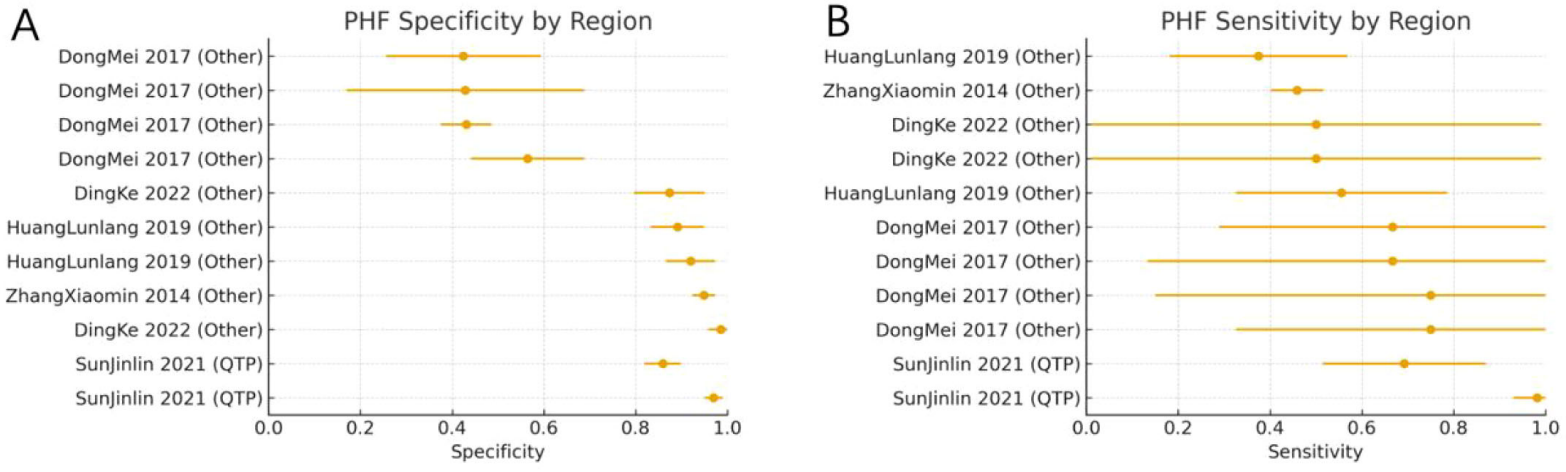
**(A)** PHF specificity by region. **(B)** PHF sensitivity by region. PHF, probability of hip fracture.

For PHF, a similar pattern was observed. In non-QTP regions, the pooled sensitivity was 0.47 (95% CI: 0.42–0.52; *I*^2^ = 0.0%; k = 9), and the pooled specificity was 0.74 (95% CI: 0.61–0.86; *I*^2^ = 98.0%; k = 9). In the Qinghai–Tibet subgroup, the pooled sensitivity increased to 0.85 (95% CI: 0.56–1.00; *I*^2^ = 90.1%; k = 2), and the pooled specificity to 0.92 (95% CI: 0.81–1.00; *I*^2^ = 95.9%; k = 2). These findings suggest better discrimination of FRAX for hip fracture in the plateau population, particularly in terms of specificity, although heterogeneity remained considerable. Forest plots are presented in **Figure 10**.

##### 3.3.2.5 Sensitivity analysis

Sensitivity analysis was conducted by sequentially excluding studies, and no study had a significant impact on heterogeneity, indicating stable results (**Figure 9B**). Similarly, removing studies with high or unclear risk of bias yielded sensitivity and specificity estimates that remained within the confidence intervals of the primary analysis, suggesting robustness of the pooled results.

##### 3.3.2.6 Fagan plot

**Figure 11:**
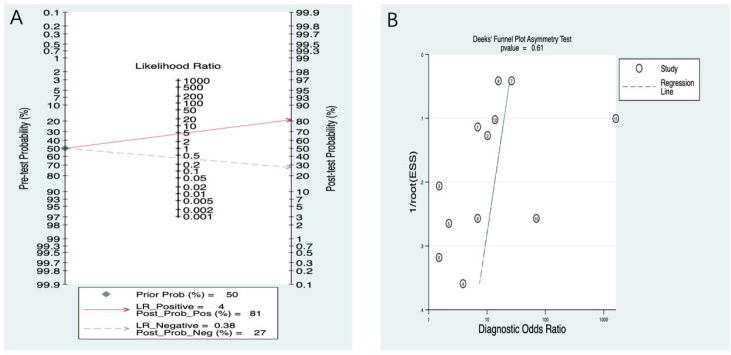
**(A)** PHF Fagan. **(B)** PHF publication bias. PHF, probability of hip fracture.

In the Fagan nomogram analysis (**Figure 11A**), the PLR was 4, and the NLR was 0.38. By connecting the pre-test probability with the values corresponding to the positive and negative likelihood ratios in the middle of the plot and intersecting with the post-test probability on the right, the post-test probability of positive and negative was obtained. Assuming that the initial correct identification probability of fracture was 50%, the probability of correct positive diagnosis through PHF modeling prediction increased from 50% to 81%.

##### 3.3.2.7 Publication bias

The publication bias test result was *p* = 0.61 (95% CI: −19.50 to 12.01), indicating no publication bias (**Figure 11B**).

#### 3.3.3 Application of FRAX high risk (PHF ≥ 3% or PMOF ≥ 20%) in fracture risk assessment in plateau population

##### 3.3.3.1 Combined effect size

**Figure 12:**
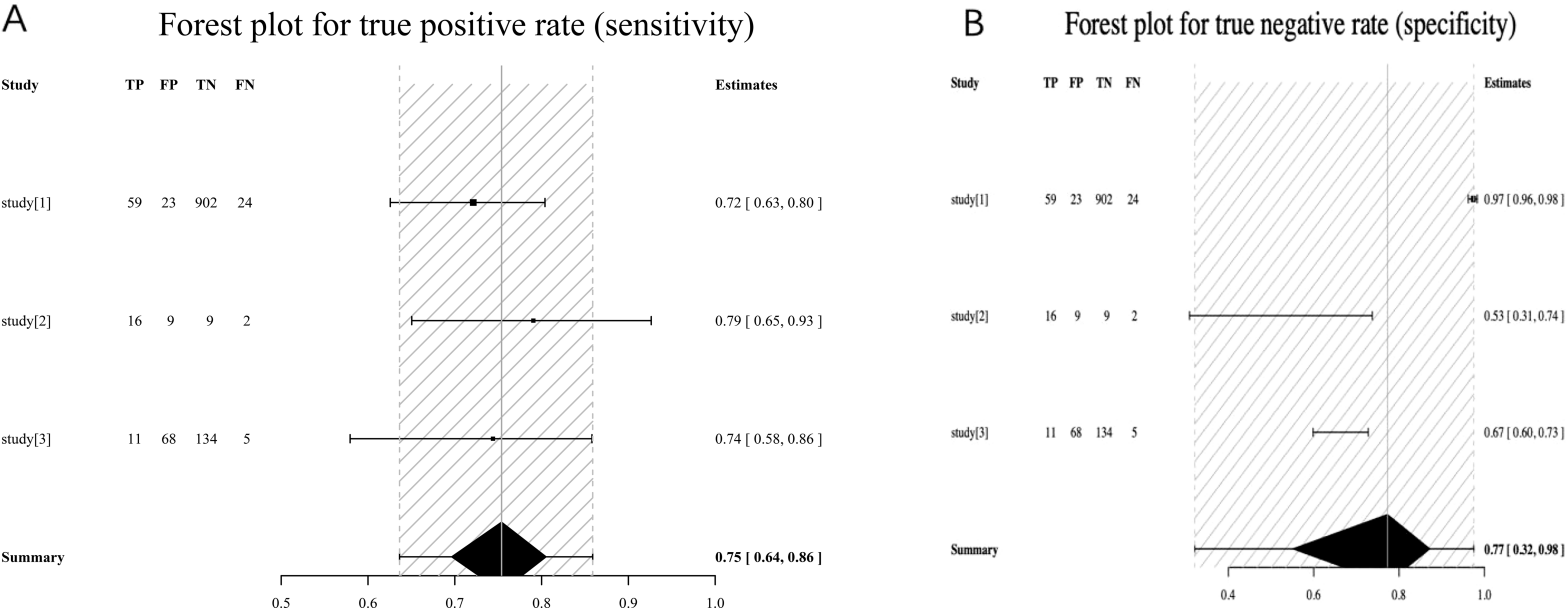
**(A)** FRAX high risk sensitivity. **(B)** FRAX high risk specificity. study[1], Zhang Juan (2019); study[2], Yan Xueping (2019); study[3], Wang Lin (2015).

A total of three studies were included. The results of Spearman’s correlation analysis showed that s = −0.50, *p* = 0.67, and there was no threshold effect, so the effect size could be combined for analysis. Considering the small number of included studies, there may be significant heterogeneity, so a random-effects model was used. After combination, compared with the gold standard of severe osteoporosis or fracture, the sensitivity of FRAX high risk was 0.75 (95% CI: 0.64–0.86), the specificity was 0.77 (95% CI: 0.32–0.98), the PLR was 15.06 (95% CI: 0.94–43.66), the NLR was 0.46 (95% CI: 0.17–1.23), and the DOR was 52.98 (95% CI: 0.75–181.44). Notably, the very wide confidence intervals—particularly for specificity (95% CI: 0.32–0.98)—indicate substantial statistical instability of these estimates, likely related to the small number of studies and heterogeneity in threshold application (**Figure 12**).

##### 3.3.3.2 SROC curve drawing

**Figure 13:**
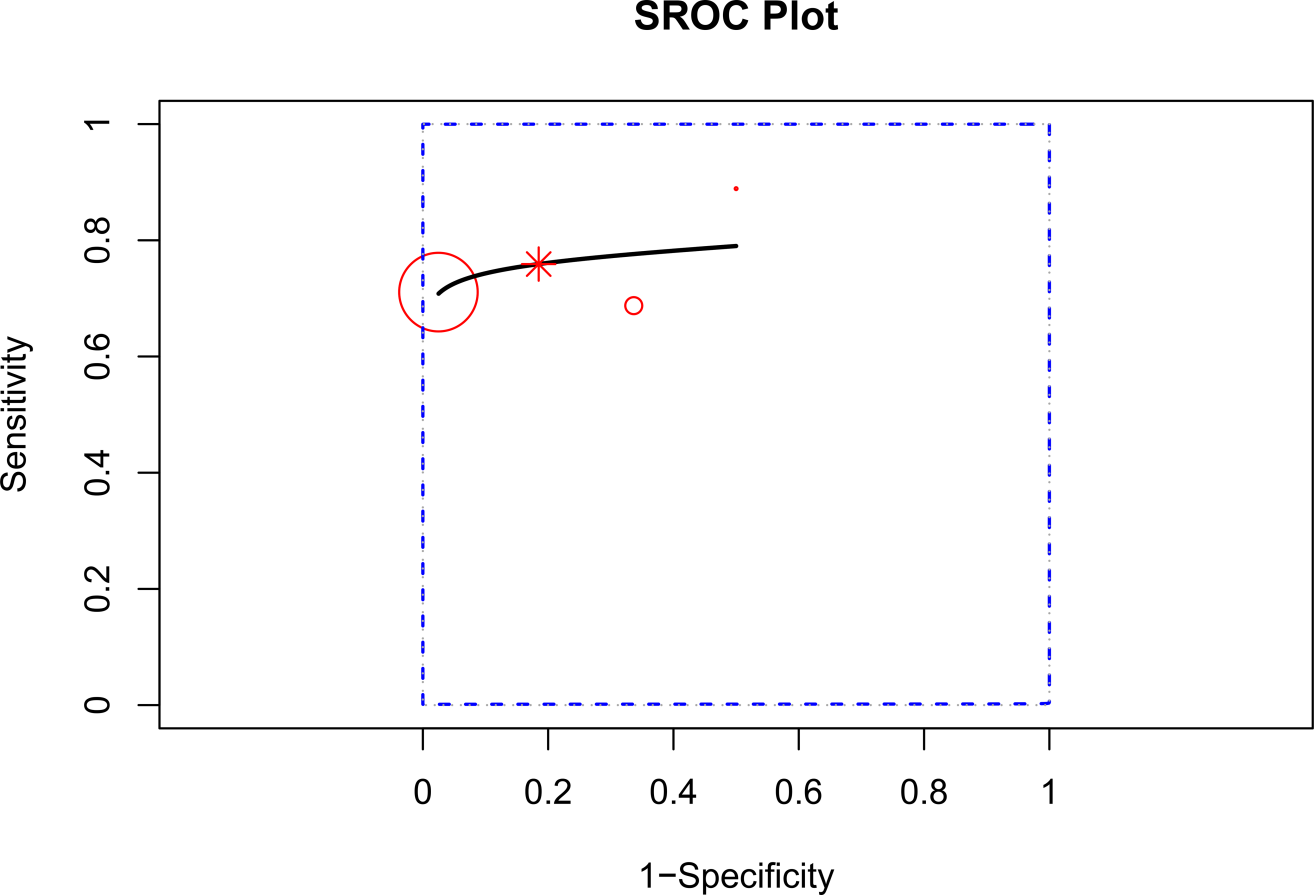
SROC curve of FRAX high risk in fracture risk assessment in plateau population. SROC, summary receiver operating characteristic.

The AUC value of FRAX high risk predicting severe osteoporosis or fracture in plateau population was 0.78 (0.08–0.98) (**Figure 13**).

## 4 Discussion

High-altitude exposure may reduce bone mineral density in adults, thereby increasing the risk of osteoporosis (10). Among postmenopausal women with diabetes residing in high-altitude regions, 27.3% of patients suffer from osteoporosis, while 42.4% have osteopenia (32). Therefore, it is particularly important to popularize the screening of osteoporosis and fracture risks in high-altitude areas. The previous risk screening strategy based on BMD has relatively few indicators, high screening costs, and difficulties in short-term promotion and application. FRAX has a certain consistency with BMD (correlation coefficient ~0.50) (33), and it is reasonable and cost-effective to popularize fracture risk screening based on FRAX in high-altitude areas in China.

Under hypoxic conditions, oxidative stress leads to the massive release of reactive oxygen species (ROS), thereby affecting bone remodeling. As a downstream factor and inducer of ROS (34, 35), hypoxia-inducible factor-1 (HIF-1α) plays a crucial role in bone remodeling and can effectively regulate the physiological activities of various cells related to the skeleton (36). Studies have shown that the low-oxygen environment in high-altitude areas induces changes in the level of hypoxia-inducible factor-1α, thereby altering bone metabolism and promoting changes in gene levels such as vascular endothelial growth factor, enhancing osteoclastic activity and inhibiting osteogenic activity, thereby affecting bone mass (37–40). Under conditions such as high-altitude hypoxia, erythropoietin (EPO) is activated and produced in the kidneys (41). Comparative studies of the bone phenotypes in polycythemia vera mouse models versus polycythemia induced by elevated circulating EPO revealed reduced bone formation and increased osteoclast numbers in both mouse types (42). Additionally, the seasonal variation in ultraviolet intensity leads to fluctuations in vitamin D levels, further affecting calcium and phosphorus metabolism (43), all of which increase the risk of osteoporotic fractures in high-altitude areas. Conversely, research on the anti-osteoporotic effects of hyperbaric oxygen therapy in rats demonstrated that hyperbaric oxygen improved bone microarchitecture (44).

To evaluate the screening performance of FRAX in high-altitude areas in China, this study has, for the first time, summarized the application results of FRAX in high-altitude areas in China through meta-analysis.

For the application of PMOF in fracture risk assessment in plateau populations, seven articles were included in the meta-analysis, and the results showed that both sensitivity and specificity had significant heterogeneity, so a random-effects model was used. After combination, the sensitivity, specificity, DOR, and SROC AUC of PMOF for severe osteoporosis or fracture were 0.70, 0.82, 10.00, and 0.82, respectively. In the Fagan nomogram analysis, the post-test probability increased from 50% to 79% through PMOF modeling prediction, and the sensitivity analysis indicated stable results, with no publication bias. This suggests that PMOF has good accuracy in assessing fracture risk in the plateau population. The sensitivity of PMOF was comparable to the results reported by Sun et al. (45) in the Han population of plain areas, but its specificity was significantly higher than that reported in ethnic minority populations, indicating that FRAX may be more advantageous in the Han population of high-altitude areas.

In addition, subgroup analyses demonstrated substantially higher specificity and moderately higher sensitivity in studies from the Qinghai–Tibet Plateau compared with other high-altitude regions, which was consistent with the directionality observed in the meta-regression. Female-only cohorts showed more stable sensitivity, whereas mixed-gender cohorts yielded higher specificity but greater variability, suggesting that both region and gender composition may act as important modifiers of FRAX diagnostic performance.

For the application of PHF in fracture risk assessment in plateau populations, five articles were included in the meta-analysis, and the results showed that both sensitivity and specificity had significant heterogeneity, so a random-effects model was used. After combination, the sensitivity, specificity, DOR, and SROC AUC of PHF for severe osteoporosis or fracture were 0.68, 0.84, 11.00, and 0.82, respectively. In the Fagan nomogram analysis, the post-test probability increased from 50% to 81% through PHF modeling prediction, highlighting its significant value in high-risk population screening. For instance, in high-altitude areas with limited medical resources, FRAX can serve as a preliminary screening tool to prioritize interventions for high-risk individuals (e.g., those with PHF ≥ 3%), thereby reducing medical costs. The sensitivity analysis indicated stable results, with no publication bias. This result is similar to the PMOF result, further suggesting that PHF has good accuracy in high-altitude areas. In clinical practice, it is recommended to combine the FRAX high-risk threshold with other indicators (e.g., history of falls) to improve diagnostic accuracy.

Similarly, subgroup analyses for PHF also showed better discrimination in plateau populations than in non-plateau regions, especially in terms of specificity. These patterns were consistent with meta-regression findings, further indicating that region, reference standard, BMD inclusion, and gender may collectively account for a substantial portion of the between-study heterogeneity.

Although the pooled sensitivity of FRAX was below 0.8, indicating moderate case-detection ability, its relatively high specificity suggests reliable identification of low-risk individuals. Therefore, FRAX should not be considered a diagnostic tool but rather a first-step screening method to stratify fracture risk. In high-altitude regions with limited access to DXA, FRAX can help prioritize individuals for further examination and targeted prevention strategies.

The AUC values for PMOF and PHF were both 0.82, indicating comparable diagnostic accuracy in predicting severe osteoporosis or fractures among high-altitude populations. This similarity suggests that both indicators may be clinically interchangeable in FRAX-based screening. The choice between PMOF and PHF in practice may depend on the local fracture epidemiology and availability of BMD testing, as PMOF incorporates broader fracture sites while PHF is more specific to hip fractures. In resource-limited settings, prioritizing the more prevalent or impactful fracture type may help optimize risk stratification.

Heterogeneity test indicates that there is significant heterogeneity between included studies, and further meta-regression and subgroup analyses were conducted to find the sources of heterogeneity, suggesting that the presence or absence of BMD, whether it is the Qinghai–Tibet Plateau area, differences in gold standards, and gender may all be causes of heterogeneity. In terms of specificity, the results of meta-regression suggest that gender and region may be the main sources of heterogeneity. The reason for this may be that the Qinghai–Tibet Plateau is the highest-altitude area in China, and some studies have reported that bone density gradually decreases with the increase of altitude (46). Fu et al. (47) and others measured the bone density values of residents living in the northwest area at altitudes of 2,800 and 1,500 m, and the results were significantly lower than the bone density values of the control group of Japanese people, indicating that the difference in altitude may be one of the main influencing factors of osteoporosis. In terms of gender, a large number of studies have shown that the bone density values of women in the same age group are significantly lower than those of men in the same age group, which may be related to the fact that men generally have a larger amount of activity, and the bone density values are increased due to the influence of mechanical load. In addition, the decrease in estrogen in women during pregnancy and perimenopause may also be one of the reasons (48, 49).

However, it is important to emphasize that the persistently high heterogeneity observed in this meta-analysis, with *I*^2^ values frequently exceeding 90%, should be regarded as a substantive limitation to inference rather than a purely statistical phenomenon.

Although subgroup and meta-regression analyses suggested that region, sex composition, BMD inclusion, and reference standards may contribute to variability in FRAX screening performance, these findings should be interpreted as exploratory and hypothesis-generating rather than as evidence of definitive effect modification. From a methodological perspective, heterogeneity likely reflects differences in study design, diagnostic thresholds, reference standards, and population characteristics across high-altitude regions. Biologically, variation in altitude exposure, ethnicity, and sex-related bone metabolism may further influence FRAX performance. Clinically, these factors limit the generalizability of pooled estimates and preclude direct translation of subgroup differences into individualized risk assessment or decision-making.

Therefore, while subgroup analyses provide useful contextual insights, the substantial residual heterogeneity underscores the need for cautious interpretation of pooled diagnostic accuracy estimates and highlights the current limitations of the available evidence.

Additionally, one included study (21) applied a T-score < −1 rather than the standard threshold for severe osteoporosis. This inconsistency may affect the comparability of the included data and introduce additional heterogeneity in the pooled diagnostic accuracy.

Furthermore, sensitivity analyses excluding studies with high or unclear risk of bias (24, 27, 30) confirmed that both PMOF and PHF pooled estimates remained within the confidence intervals of the main analyses, indicating that methodological limitations of individual studies were not the primary drivers of the observed heterogeneity.

However, numerical stability should not be interpreted as high evidential certainty, as many of the included studies were affected by methodological limitations, and conclusions based on such evidence warrant cautious interpretation. The methodological limitations inherent to unpublished theses and reports—such as incomplete reporting, lack of peer review, and variable study rigor—inevitably reduce the overall credibility of the evidence base, regardless of numerical stability in sensitivity analyses.

There are few studies on the application of FRAX high risk (PHF ≥ 3% or PMOF ≥ 20%) in fracture risk assessment in plateau population, and this study only included three articles. A random-effects model was directly used, and the sensitivity, specificity, DOR, and SROC AUC after combination were 0.75, 0.77, 52.98, and 0.78, respectively. This result is only higher than PMOF and PHF in terms of sensitivity, but it does not have advantages in other aspects. Notably, the confidence interval for specificity was extremely wide (95% CI: 0.32–0.98), indicating marked statistical instability. This instability likely reflects the small number of available studies as well as heterogeneity in how the high-risk threshold was operationalized across cohorts. Moreover, the FRAX high-risk threshold was originally designed for treatment decision-making rather than diagnostic classification, further limiting the interpretability of these findings. Therefore, conclusions regarding the diagnostic value of FRAX high-risk thresholds in plateau populations should be considered exploratory.

It is important to note that FRAX was designed as a screening and fracture risk estimation tool, not as a standalone diagnostic instrument. The presence of osteoporosis still requires confirmation by BMD testing using DXA when available, and FRAX should be interpreted as an adjunct to, rather than a substitute for, BMD-based assessment. Clarifying this distinction is essential to avoid misinterpreting FRAX as a diagnostic alternative in clinical practice. It should be emphasized that FRAX thresholds were originally developed to guide treatment decisions rather than to serve as diagnostic cut-offs. Applying these thresholds in a diagnostic-accuracy framework introduces a conceptual mismatch, which limits the interpretation of FRAX performance and precludes diagnostic-level claims.

The limitations of this study include the following: 1) most included studies used a cross-sectional design, which precludes the validation of FRAX’s longitudinal predictive efficacy. 2) The included studies excluded some articles that could not obtain data, which may lead to result bias. 3) There is significant heterogeneity between different studies, and different altitude areas and different measurement methods may also lead to result bias. 4) This study did not analyze the best threshold, which needs to be further supplemented in future studies. 5) FRAX does not cover risk factors specific to high altitudes (e.g., vitamin D deficiency and physical activity patterns), and future studies need to develop localized models.

Overall, while FRAX shows a certain degree of screening performance in high-altitude populations, the strength of evidence supporting its use remains limited. The substantial heterogeneity, wide confidence intervals, and reliance on a small number of studies indicate that the current findings should be viewed as exploratory rather than confirmatory, and they do not support definitive conclusions regarding clinical application.

## Data Availability

The original contributions presented in the study are included in the article/supplementary material. Further inquiries can be directed to the corresponding author.

## References

[1] Johnston CB Dagar M . Osteoporosis in older adults. Med Clin North Am. (2020) 104:873–84. doi: 10.1016/j.mcna.2020.06.004, PMID: 32773051

[2] Lorentzon M . Treating osteoporosis to prevent fractures: current concepts and future developments. J Intern Med. (2019) 285:381–94. doi: 10.1111/joim.12873, PMID: 30657216

[3] El Miedany Y . FRAX: re-adjust or re-think. Arch Osteoporos. (2020) 15:150. doi: 10.1007/s11657-020-00827-z, PMID: 32989561 PMC7522068

[4] Vandenput L Johansson H McCloskey EV Liu E Åkesson KE Anderson FA . Update of the fracture risk prediction tool FRAX: a systematic review of potential cohorts and analysis plan. Osteoporos Int. (2022) 33:2103–36. doi: 10.1007/s00198-022-06435-6, PMID: 35639106

[5] Chinese Society of Osteoporosis and Bone Mineral Research . Guidelines for the diagnosis and treatment of primary osteoporosis (2022). Chin Gen Pract. (2023) 26:1671–91. doi: 10.12114/j.issn.1007-9572.2023.0121

[6] Du GC Qin JL AD Li QZ Meng GY . Analysis of factors affecting fracture healing in high-altitude areas. Guangdong Med. (2016) 37:1823–6. doi: 10.13820/j.cnki.gdyx.2016.12.011

[7] Yang J Tang K Gao M Fan HH Wang W Liu XL . Survey of quantitative CT bone density and osteoporosis prevalence in middle-aged and elderly people in kunming area. Chin J Osteoporos. (2022) 28:1330–5. doi: 10.3969/j.issn.1006-7108.2022.09.015

[8] Wang H Ma Q Ma BA . Research status of osteoporosis in high-altitude areas of China. Med Inf. (2020) 33:34–7. doi: 10.3969/j.issn.1006-1959.2020.23.011

[9] Qiao Y Zheng H Cheng R Rong L Guo J Li G . A review of the relationship between gut microbiota and osteoporosis in high altitude environments. J Health Popul Nutr. (2025) 44:241. doi: 10.1186/s41043-025-00994-0, PMID: 40624713 PMC12235887

[10] Zuo H Zheng T Wu K Yang T Wang L Nima Q . China Multi-Ethnic Cohort (CMEC). High-altitude exposure decreases bone mineral density and its relationship with gut microbiota: Results from the China multi-ethnic cohort (CMEC) study. Environ Res. (2022) 215:114206. doi: 10.1016/j.envres.2022.114206, PMID: 36058270

[11] Wang S Zhang F Guo Y Zhang C Zhong Y Hao D . Exposure to high altitude is associated with an elevated risk of hip fracture: a retrospective cohort study using data from the CHARLS. BMC Geriatr. (2025) 25:884. doi: 10.1186/s12877-025-06492-6, PMID: 41214560 PMC12604388

[12] Camacho-Cardenosa M Camacho-Cardenosa A Timón R Olcina G Tomas-Carus P Brazo-Sayavera J . Can hypoxic conditioning improve bone metabolism? A systematic review. Int J Environ Res Public Health. (2019) 16:1799. doi: 10.3390/ijerph16101799, PMID: 31117194 PMC6572511

[13] Da W Tao L Zhu Y . The role of osteoclast energy metabolism in the occurrence and development of osteoporosis. Front Endocrinol (Laus). (2021) 12:675385. doi: 10.3389/fendo.2021.675385, PMID: 34054735 PMC8150001

[14] Brent MB . A review of the skeletal effects of exposure to high altitude and potential mechanisms for hypobaric hypoxia-induced bone loss. Bone. (2022) 154:116258. doi: 10.1016/j.bone.2021.116258, PMID: 34781048

[15] Zhu SY Shi ZY Chen YL Wu YH . Effects of high altitude exposure on human bone. J Med Inf. (2025) 38:178–82. doi: 10.3969/j.issn.1006-1959.2025.08.038

[16] Kim JH Yoon SM Song SU Park SG Kim WS Park IG . Hypoxia suppresses spontaneous mineralization and osteogenic differentiation of mesenchymal stem cells via IGFBP3 up-regulation. Int J Mol Sci. (2016) 17:1389. doi: 10.3390/ijms17091389, PMID: 27563882 PMC5037669

[17] Kondo T Otsuka Y Aoki H Goto Y Kawaguchi Y Waguri-Nagaya Y . The inducible nitric oxide synthase pathway promotes osteoclastogenesis under hypoxic culture conditions. Am J Pathol. (2021) 191:2072–9. doi: 10.1016/j.ajpath.2021.08.014, PMID: 34560064

[18] Murray AJ Montgomery HE Feelisch M Grocott MPW Martin DS . Metabolic adjustment to high-altitude hypoxia: from genetic signals to physiological implications. Biochem Soc Trans. (2018) 46:599–607. doi: 10.1042/BST20170502, PMID: 29678953

[19] West JB . High-altitude medicine. Am J Respir Crit Care Med. (2012) 186:1229–37. doi: 10.1164/rccm.201207-1323CI, PMID: 23103737

[20] Huang QX Huang XW . QUADAS-2 tool for quality assessment in diagnostic meta-analysis. Ann Palliat Med. (2022) 11:1844–5. doi: 10.21037/apm-22-204, PMID: 35400153

[21] Cai ST Sun W Liu H . Clinical significance of fracture risk assessment tool (FRAX) in evaluating postmenopausal women’s bone density. Chin J Osteoporos. (2017) 23:177–82. doi: 10.3969/j.issn.1006-7108.2017.02.009

[22] Zhang J . Applicability evaluation of fracture risk assessment tool in type 2 diabetes population in xinjiang. Xinjiang: Xinjiang Med University (2019).

[23] Huang LL Wang L Wang SY Yin WJ Sun ZM Li MX . Discussion on the clinical application value of fracture risk assessment tool FRAX for tibetan patients. Chin J Osteoporos. (2019) 25:85–8. doi: 10.3969/j.issn.1006-7108.2019.01.015

[24] Dong M Jin SX Han XM . Clinical research on fracture risk assessment using FRAX fracture risk prediction tool in hohhot population. Chin J Osteoporos. (2017) 23:1067–70. doi: 10.3969/j.issn.1006-7108.2017.08.019

[25] Yan XP Zhou L Chen L Xiang T Han M . Preliminary study on osteoporosis screening in postmenopausal women with maintenance hemodialysis. West China Med J. (2019) 34:764–8. doi: 10.7507/1002-0179.201905244

[26] Cao DG Zhang SL Yang FB Shen K . Clinical research on predicting osteoporotic vertebral fractures in postmenopausal women using FRAX. Chin J Osteoporos. (2020) 26:273–7. doi: 10.3969/j.issn.1006-7108.2020.02.024

[27] Zhang XM . Clinical research on assessing the risk of osteoporotic hip fractures in middle-aged and elderly people in taiyuan city using FRAX. Shanxi: Shanxi Med University (2014).

[28] Wang L . Research on the correlation between FRAX, BMD and osteoporotic fractures in women in kunming area. Kunming: Kunming Med University (2015).

[29] Ding K . Clinical research on the applicability of FRAX combined with DXA in assessing the risk of osteoporotic fractures in patients with maintenance hemodialysis. Guangxi Zhuang Autonomous Region: Youjiang Med University Nationalities (2022).

[30] Zhang XJ Sun Y Li Y Cui XY Li JF Liu W . Research on the predictive effect of FRAX combined with DXA on fractures in patients with osteoporosis. J Chongqing Med Univ. (2019) 44:1171–5. doi: 10.13406/j.cnki.cyxb.002141

[31] Sun JL . Clinical research on predicting fracture risk in patients with type 2 diabetes in qinghai area using FRAX. Qinhai: Qinghai University (2021).

[32] Zhou L Song J Yang S Meng S Lv X Yue J . Bone mass loss is associated with systolic blood pressure in postmenopausal women with type 2 diabetes in Tibet: a retrospective cross-sectional study. Osteoporos Int. (2017) 28:1693–8. doi: 10.1007/s00198-017-3930-6, PMID: 28154942

[33] LeBoff MS Greenspan SL Insogna KL Lewiecki EM Saag KG Singer AJ . The clinician’s guide to prevention and treatment of osteoporosis. Osteoporos Int. (2022) 33:2049–102. doi: 10.1007/s00198-021-05900-y, PMID: 35478046 PMC9546973

[34] Dosek A Ohno H Acs Z Taylor AW Radak Z . High altitude and oxidative stress. Respir Physiol Neurobiol. (2007) 158:128–31. doi: 10.1016/j.resp.2007.03.013, PMID: 17482529

[35] Bai XC Lu D Bai J Zheng H Ke ZY Li XM . Oxidative stress inhibits osteoblastic differentiation of bone cells by ERK and NF-kappaB. Biochem Biophys Res Commun. (2004) 314:197–207. doi: 10.1016/j.bbrc.2003.12.073, PMID: 14715266

[36] Meng X Wielockx B Rauner M Bozec A . Hypoxia-inducible factors regulate osteoclasts in health and disease. Front Cell Dev Biol. (2021) 9:658893. doi: 10.3389/fcell.2021.658893, PMID: 33816509 PMC8014084

[37] Chen L Xi AQ A XR Li ZA Xi K Peng H . Changes and correlations of serum bone metabolism biochemical indicators, chemerin, and hypoxia-inducible factor 1α in han elderly males living in high-altitude areas. Chin J Tissue Eng Res. (2016) 20:4876–82. doi: 10.3969/j.issn.2095-4344.2016.33.002

[38] Li DC . Correlation study of serum HIF-1a, HIF-2a, VEGF and bone metabolism indicators in elderly males with osteoporosis in high-altitude areas. Chin J Osteoporos. (2015) 21:1328–32.doi: 10.3969/j.issn.1006-7108

[39] Ma JW Li DC Zhang ZG Li Y Wang YB Cao ZQ . Correlation of oxidative stress-related factors and bone metabolism indicators in elderly males with degenerative osteoporosis in high-altitude areas: A non-randomized controlled clinical trial protocol. Chin J Tissue Eng Res. (2017) 21:5103–7. doi: 10.3969/j.issn.2095-4344.2017.32.004

[40] Li M Li J Fu K . Effects of total flavonoids of drynariae rhizoma on the expression of HIF-1α and VEGF in knee osteoarthritis model rabbits. China Pharm. (2018) 29:2484–8. doi: 10.6039/j.issn.1001-0408.2018.18.09

[41] Tsiftsoglou AS . Erythropoietin (EPO) as a key regulator of erythropoiesis, bone remodeling and endothelial transdifferentiation of multipotent mesenchymal stem cells (MSCs): implications in regenerative medicine. Cells. (2021) 10:2140. doi: 10.3390/cells10082140, PMID: 34440909 PMC8391952

[42] Oikonomidou PR Casu C Yang Z Crielaard B Shim JH Rivella S . Polycythemia is associated with bone loss and reduced osteoblast activity in mice. Osteoporos Int. (2016) 27:1559–68. doi: 10.1007/s00198-015-3412-7, PMID: 26650379 PMC5319412

[43] Liu WX Xu T Wang C Di JZ . Clinical research progress on osteoporosis and fracture risk in high-altitude areas of China. Chin J Trauma Orthop. (2019) 21:545–8. doi: 10.3760/cma.j.issn.1671-7600.2019.06.015

[44] Imerb N Thonusin C Pratchayasakul W Chanpaisaeng K Aeimlapa R Charoenphandhu N . Hyperbaric oxygen therapy exerts anti-osteoporotic effects in obese and lean D-galactose-induced aged rats. FASEB J. (2023) 37:e23262. doi: 10.1096/fj.202301197RR, PMID: 37855727

[45] Sun J Yang MX Zhang ZH . Research on the application results of FRAX assessment tool in han and ethnic minority populations in China. Chin J Osteoporos. (2021) 27:1147–53. doi: 10.3969/j.issn.1006-7108.2021.08.010

[46] Ma DQ Gao SY Yang C . Clinical research on osteoporosis in high-altitude areas. Plateau Med J. (1994) 1:28–32.

[47] Fu C Liu XY Ge BF Bai MH Song Y . Study on bone density of residents in northwest plateau area. Chin J Trauma. (2002) 4:11–4. doi: 10.3969/j.issn.1003-0034.2002.04.003

[48] Lei XD Yu H Long Q Li JW Dai B . Research progress on the pathogenesis of postmenopausal osteoporosis. Chin J Osteoporos. (2021) 27:1681–4. doi: 10.3969/j.issn.1006-7108.2021.11.024

[49] Jiang JK Song XY . Correlation study on reproductive characteristics and bone density in postmenopausal women. Chin J Osteoporos. (2019) 25:330–333+365. doi: 10.3969/j.issn.1006-7108.2019.03.006

